# Coronavirus disease (COVID-19) – impact on vaccine preventable diseases

**DOI:** 10.2807/1560-7917.ES.2020.25.18.2000756

**Published:** 2020-05-07

**Authors:** Daniel Hungerford, Nigel A Cunliffe

**Affiliations:** 1 The Centre for Global Vaccine Research, Institute of Infection and Global Health, University of Liverpool, members of Liverpool Health Partners, Liverpool, United Kingdom.

**Keywords:** vaccine-preventable diseases, vaccine, immunisation, Covid-19, surveillance, outbreak

To the editor: We read with interest the short notice about the launch of the European Vaccination Information Portal in conjunction with the European Immunization Week 2020 [1]. We believe this launch comes at a critical time, as the response to coronavirus disease (COVID-19) consumes government, public health and clinical resources. But what does this mean for vaccine preventable diseases (VPDs)?

Many countries with confirmed COVID-19 cases initiated ‘lockdown’ as a response to the pandemic, with implementation of strict social distancing, isolation and quarantine. This will likely reduce community transmission of many VPDs. But lockdown will also present a huge challenge for general practitioners (GPs) and community healthcare to deliver immunisations to 2020 birth cohorts, initiate catch-up campaigns with older cohorts and deliver immunisations to at-risk groups. Household isolation and COVID-19 illness in families with new-born children, combined with disruption to vaccine supply, healthcare staffing shortages and enhanced infection prevention procedures, are likely to significantly reduce opportunities for timely delivery of routine immunisations.

Uptake of vaccines in Europe has continued to decline in recent years, including in the United Kingdom (UK) [2]. The fall in MMR vaccine coverage has been followed by large outbreaks of measles, with more than 500,000 confirmed cases globally in 2019, more than in any single year since 2006 [3,4]. While spring and summer 2020 are anticipated to be accompanied by a lower incidence of many VPDs, possible further declines in routine vaccine uptake in 2020 birth cohorts may surpass recent years, and thus generate a record number of susceptible children. If lockdown ends, social distancing relaxes and formal schooling returns before, or during the 2020/21 autumn and winter season, then outbreaks of diseases such as measles, pertussis, and rotavirus gastroenteritis appear inevitable.

While governments, healthcare professionals and researchers are rightly focusing on the immediate response to the COVID-19 pandemic, we must ensure that sufficient resource and consideration is given to delivery of routine vaccinations. This has been highlighted in the UK by National Health Service England and Public Health England, who have recommended GP practices continue with routine immunisation services without delays [5]. This is clearly the appropriate public health approach, but the practicalities of delivering immunisations under the strain of the COVID-19 outbreak will inevitably lead to unintended drops in vaccine coverage.

In order to rapidly identify hotspots of falling uptake, immunisation rates should be monitored by analysts at the macro- (administrative area) and micro- (GP or neighbourhood level) level. The latter is critical since we know that the most-deprived populations are disproportionately affected by both immunisation service disruption and by disease burden [6]. The UK Parliament’s Knowledge Exchange Unit has created a COVID-19 Outbreak Expert Database, which is intended to enable researcher access to facilitate a rapid response to COVID-19 and its impacts.

Planning for return to pre-pandemic functionality with more relaxed restrictions on social distancing must not only consider COVID-19 transmission but also the ability of the health service to cope with more endemic infection challenges. This decision should be informed by evidence from vaccine coverage surveillance, modelling and expert information from GPs and immunisation teams. This will help ensure coverage in infant cohorts has reached a threshold whereby the risk of VPD outbreaks are acceptably low. The world cannot afford a legacy of this pandemic to be increased disability and mortality from VPDs.
